# The association between diet quality and chronic obstructive pulmonary disease: a case-control study

**DOI:** 10.1186/s12889-023-16586-8

**Published:** 2023-10-23

**Authors:** Batoul Ghosn, Shokouh Onvani, Mohammad Emami Ardestani, Awat Feizi, Leila Azadbakht, Ahmad Esmaillzadeh

**Affiliations:** 1 Department of Community Nutrition, School of Nutritional Sciences and Dietetics, Tehran University of Medical Sciences, P.O. Box 14155-6117, Tehran, Iran; 2 Food Security Research Center, Isfahan University of Medical Sciences, Isfahan, Iran; 3 Department of Community Nutrition, School of Nutrition and Food Science, Isfahan University of Medical Sciences, Isfahan, Iran; 4 Department of Internal Medicine, Division of Pulmonology), School of Medicine, Isfahan University of Medical Sciences, Isfahan, Iran; 5 Department of Epidemiology and Biostatistics, Endocrinology and Metabolism Research Center, Isfahan University of Medical Sciences, Isfahan, Iran; 6 Obesity and Eating Habits Research Center, Endocrinology and Metabolism Molecular- Cellular Sciences Institute, Tehran University of Medical Sciences, Tehran, Iran

**Keywords:** COPD, HEI-2010, Healthy eating

## Abstract

**Background:**

Previous investigations have primarily examined the relationship between various dietary patterns and the risk of chronic obstructive pulmonary disease (COPD); however, there have been limited studies that have evaluated the association between diet quality presented by Healthy Eating Index 2010 (HEI-2010) and COPD. The aim of this study was to investigate this association in Iranian population.

**Methods:**

This case-control study recruited 84 cases and 252 healthy controls who were randomly selected. Diet, smoking, and physical activity were assessed using validated questionnaires. The HEI-2010 score ranged from zero to hundred twenty, with zero indicating an unhealthy diet and hundred twenty indicating a healthy diet. Logistic regression models were utilized to analyze the association between HEI-2010 and the odds of COPD.

**Results:**

Results from logistic regression showed that individuals with higher HEI scores had a significantly lower odds of COPD (OR: 0.34; 95% CI: 0.16–0.72). After adjusting for confounders, individuals with the highest HEI score were 82% less likely to have COPD (OR: 0.18; 95% CI: 0.03–0.96). This association remained significant after adjusting for smoking and physical activity (OR: 0.08; 95% CI: 0.01–0.93) and with additional adjustment for BMI (OR: 0.08; 95% CI: 0.01–0.92).

**Conclusions:**

This study found a significant association between a higher HEI-2010 score and a lower odd of COPD in the Iranian population. These results suggest that a healthy diet may play a crucial role in reducing the odds of COPD and in improving the function of the lungs. However, further prospective studies are warranted to elucidate this relationship.

**Supplementary Information:**

The online version contains supplementary material available at 10.1186/s12889-023-16586-8.

## Background

COPD is a major global health issue that causes a significant burden on countries worldwide. According to the World Health Organization (WHO), Chronic obstructive pulmonary disease (COPD) is the third leading cause of death worldwide, causing 3.23 million deaths in 2019 [1]. It is described as progressive and poorly reversible disease of the lungs with significant morbidity and mortality [2]. Incidence of COPD is increasing due to prevalence of smoking and advanced age [3]. The worldwide mean prevalence of COPD is 13.1%, but the rate differs significantly among countries and populations [4]. Patients with COPD are at increased risk of developing pneumonia [5], lung cancer [6], and cardiovascular diseases [7], among others. The economic burden of COPD in USA reached $32.1 billion in 2010, and have increased to 49 billion in 2020 [8].

Although smoking is the leading cause of impaired lung function, several other factors, including diet, contribute to COPD incidence [9]. In terms of diet, an inverse association between higher dietary intake of fiber, fruit and vegetables, and fish and lower risk of COPD have been found in previous studies [10–12]. However, examining the link between whole diet and chronic conditions can provide additional information. The Healthy Eating Index (HEI) is a measure of whole diet quality that assesses the adherence to the dietary guidelines [13]. The association between HEI and risk of several conditions, including obesity [14], depression [15], diabetes [16], and mortality [17] was examined in earlier publications. While previous studies have found a protective association between a prudent dietary pattern (rich in whole grains, fish, vegetables, and fruits) and COPD [18–20], no study has yet examined the relationship between HEI-2010 and COPD, despite several dietary factors being associated with COPD in previous publications [21–23].

It must be kept in mind that the associations between dietary intakes and risk of COPD were mainly examined in western countries [24, 25] and limited information are available in this regard from developing nations [26]. This is particularly relevant when the dietary intakes of people in developing countries, especially those in the Middle East, are significantly different from those in western countries [27]. For instance, people in the Middle East are taking a higher percent of their energy from carbohydrates [28], which are mostly from refined sources. In addition, they are experiencing a nutrition transition, moving from their healthy traditional diets to non-healthy western diets [29]. There is evidence that the spread of Western diet and lifestyle worldwide has led to an increase in the availability and affordability of foods associated with a Western diet in developing countries [30]. Also, prevalence of factors contributing to COPD might be geographically different. Smoking [31], physical activity [32] and air pollution [33] may differ between regions and countries. Therefore, examining the association between diet and COPD in specific regions can provide valuable insights into the diet-disease link. In this study, we aimed to assess the association between adherence to the HEI-2010 and odds of COPD in a specific region of Iran. While our findings may not be generalizable to the entire Iranian population, they provide important information about the relationship between diet and COPD in this particular region.

## Methods and materials

### Participants

This case-control study was done in Al-Zahra University Hospital Isfahan, Iran, between 2015 and 2016. Sample size was calculated using type 1 error as 5% and study power of 90%. The ratio of control to cases was considered as 3 to 1 and the odds ratio of COPD among those in the highest score of HEI-2010 was compared to those in the lowest score and was considered as 1.5. The common ratio was considered as 0.25 for those who were at the highest score of HEI-2010. Using this information, we reached 84 cases and 252 controls. Cases were randomly selected from the hospital database using a computer-generated random number sequence. Random sampling was used to ensure that the cases were representative of the population of interest and to minimize selection bias. 84 newly diagnosed COPD patients were recruited in Al-Zahra University hospital in Isfahan, Iran. Cases in our study were patients over the age of 30 who had been diagnosed with COPD by a pulmonologist. The diagnosis was based on the results of a spirometry test, which measures lung function. Specifically, patients were considered to have COPD if their forced expiratory volume in 1 s (FEV1) divided by their forced vital capacity (FVC) was less than 70%, or if their FEV1 was less than 80% of the predicted value [34]. The control group consisted of 252 healthy controls without a history of COPD from the same hospital. Cases and controls were matched in terms of age (range 5 years) and sex. Controls were subjects at the same hospital as the cases who attended outpatients’ clinics and were recruited at the same time as the cases. Inclusion criteria for cases was being diagnosed with COPD based on physician diagnosis and spirometry test. We excluded participants if they had a history of a stroke, dementia, or any health condition that would prevent them from an interview. Also, individuals having renal failure, inflammatory bowel disease, long time treatment by steroids drugs, severe heart failure, chronic infections (tuberculosis, HIV etc.), chronic liver cirrhosis, cachexia, uncontrolled thyroid disease, rheumatoid arthritis, cancer in the past 3 years, and other pulmonary problems were not included in the study due to their impact on the ability to respond to the interview or their effect on the patients’ dietary pattern. Finally, all 336 participants participated in the study (supplementary figure). To control selection bias in our study, adjustment for the matching variables (age and sex) were done in the logistic regression analysis and “balanced matching” was applied to improve test power [35]. Also, we have carefully selected our controls from the same source population as our cases and have ensured that they are representative of the exposure distribution in the source population. Additionally, we have used stratification and other statistical methods to control potential confounding factors that could have caused selection bias. The whole project was approved by institutional review board (IRB) of Isfahan University of Medical Sciences, Isfahan, Iran. All participants have signed an informed consent.

### Dietary intakes assessment

Assessment of usual dietary intakes of participants over the past year was done using 168-item semiquantitative FFQ where its validity and reliability was mentioned previously [36]. The FFQ included 168 food items where each food item had a standard serving size commonly consumed by Iranians. Face-to-face interview was done by a trained interviewer. All study subjects were requested to report their consumption frequency of a given food item in the previous year on a daily, weekly, or monthly basis considering the serving size given for each food item. Reported frequency consumptions were converted to grams per day using household measures by multiplying the frequency of each food to its specific serving size [37]. To compute nutrient intakes from the FFQ, we used the Nutritionist IV software [38, 39], which was modified for Iranian foods. This software allowed us to accurately estimate energy, macronutrient, micronutrient, and HEI-2010 food group intakes for each participant based on their reported consumption frequencies. Energy intake was computed by summing the caloric content of all reported foods. Macronutrient intakes were calculated by summing the intakes of carbohydrates, proteins, and fats from all reported foods. Micronutrient intakes were estimated by summing the intakes of vitamins and minerals from all reported foods. Finally, HEI-2010 food group intakes were measured by classifying each reported food item into one of the HEI-2010 food groups and summing the intakes of all foods within that group.

### Construction of HEI-2010

Assessment of diet was done using the HEI-2010 index considered as a reference for a healthy diet [40]. It’s composed of 12 food groups: fruits (including fruit juice and whole fruit), refined grains, vegetables, whole grains, dairy, beans and greens, protein foods, plant proteins and sea food, fatty acids, empty calories, and sodium). No data about alcohol intake was assessed in this study. To construct HEI-2010 score, we used the residual method to calculate the intake of all food groups after adjusting for energy intake. Then we categorized participants according to the decile categories of these food components. In each of the 12 HEI-2010 food groups, decile categories were made. Participants have the highest score of 10 when they were in the highest decile category of the healthy food group (i.e., fruits, whole grains, vegetables, nuts and legumes, long chain omega-3 fats and polyunsaturated fatty acids), while they scored the lowest score of 0 when they were in the lowest decile category of these food groups. Regarding the unhealthy food groups (i.e., red and processed meat, sugar sweetened drinks, fruit juice, sodium intake, trans fat, saturated fatty acids and added sugars), a score of 10 was given for participants in the lowest decile category of these food groups, whereas a score of zero was given for participants on the highest deciles of these food groups. Participants in the deciles 9 to 2 (counting downwards) of these food items took the scores of 2 to 9 (counting upwards) respectively. Finally, to determine HEI-2010 score, scores were summed up to reach a score ranging from zero to hundred twenty.

### Assessment of pulmonary function

A trained technician assessed the lung function using spirometry testing and FEV1, FVC, and FEV1/FVC were acquired. Some important respiratory symptoms include breathlessness, sputum production and chronic cough. Chronic cough is characterized as coughing for greater than 3 weeks [41]. Sputum production refers to production of sputum for more than 3 months in 2 consecutive years [42, 43]. Breathlessness is described using a visual analogue scale which is a 100-mm horizontal line with clarifying words on both sides to help individuals elaborate about their breathlessness rate using graphical observation [44]. Shortness of breath was measured using a spirometry test.

### Assessment of other variables

Data on socio-demographic variables that included information about age, education level (illiterate, elementary, high school, diploma, university), marital status (single, married, widow, divorced), gender, socioeconomic status (owning a car, owning a home and employment) and family history of pulmonary disorders were collected using a questionnaire. Weight was measured with individuals in minimal cloth with no shoes to the nearest 100 g. Height was carried out with individuals’ bare feet and with relaxed shoulders. Body mass index (BMI) was computed as weight (kg) divided by height (m^2^). Physical activity was evaluated by the long form of International Physical Activity Questionnaire and expressed as MET-h/wk where its validity was mentioned elsewhere [45, 46]. Information about cigarette smoking (active smoker (smoking at least one cigarette a day) or using waterpipe (i.e. hookah), ex-smoker (someone who has smoked more than 100 cigarettes in their lifetime but has not smoked in the last 28 days) and non-smoker (not smoking tobacco), as well as passive smoking (involuntary inhaling of smoke from other people’s cigarettes, cigars, or pipes)) were also collected using a pre-tested self-administered questionnaire. The smoking questionnaire used in our study was pre-tested to ensure its validity and reliability. This involved administering the questionnaire to a small sample of participants and analyzing their responses to identify any issues with the questions or response options. Based on the results of the pre-test, revisions were made to the questionnaire to improve its clarity and accuracy [47].

### Statistical methods

For continuous variables independent sample Student’s t test was used, whereas for categorical variables the chi-square test. The dietary intakes and general characteristics were also assessed across categories of the HEI-2010 score using ANOVA test (one-way analysis of variance) applied for continuous variables while chi-square test applied for categorical variables. Conditional logistic regression was fitted to assess the association between HEI-2010 score and COPD by using multi-models. Gender (male/female), residence (urban/ industrial/rural), age (continuous), relationship status (married/single/divorced), social economic status (SES) (employment, owning a car/home), education level (illiterate, elementary, high school, diploma, university), family history of COPD (yes/no) were adjusted for in the first model. Physical activity and smoking were additionally adjusted in the second model. We adjusted further for body mass index (BMI) in the third model. Confounders were selected in reference to earlier publications. The IBM SPSS statistics 25 program was used for the statistical analyses carried in this study. Level of significance was measured at *p* < 0.05.

## Results

Table 1 shows descriptive characteristics of cases and control. Cases were more likely to have a positive pulmonary history and less likely to be physically active, educated, employed, married, and owning a car or a home. In terms of smoking status, cases were more likely to be active, passive, non-smokers or using water pipe, while controls were more likely to be previous smokers. No other significant results were reached in respect to other general characteristics of cases and control. As for the distribution of study participants in terms of general characteristics across quartiles of HEI-2010 score, controls in the highest quartiles were more likely to be older, employed, owning a car or a home, physically active compared to those in the lowest quartile. As for cases, subjects in the highest quartile of HEI-2010 score were less likely to be married and more likely to be educated, owning a car or a home compared to those in the lowest quartile. In terms of smoking status, cases in the highest quartile of HEI-2010 score were more likely to be active smokers while less likely to be non-smokers compared to those in the lowest quartile. As for controls, subjects in the highest quartile of HEI-2010 score were less likely to be active smokers, ex-smokers or using waterpipe while more likely to be non-smokers or passive smokers. No other significant results were reached in terms of general characteristics across quartiles of HEI-2010 score.

**Table 1:** General characteristics of study participants

Characteristics			Cases (*n *= 84)	Control (*n* = 252)	*P*-value	Quartiles of HEI-2010 score									
						Cases					Control				
						Q1 (*n* = 27)	Q2 (*n* = 16)	Q3 (*n* = 16)	Q4 (*n* = 25)	*P*-value	Q1 (*n* = 57)	Q2 (*n* = 68)	Q3 (*n* = 68)	Q4 (*n* = 59)	*P*-value
Age, y			57 ± 12	55 ± 12	0.2	57 ± 13	59 ± 10	55 ± 15	56 ± 12	0.86	52 ± 13	52 ± 11	59 ± 12	56 ± 11	**0.004**
Gender		Male	75 (89)	225 (89)	1	24 (32)	14 (18)	13 (17)	24 (32)	0.51	47 (21)	64 (28)	63 (28)	51 (23)	0.12
		Female	9 (11)	27 (11)	1	3 (33)	2 (22)	3 (33)	1 (11)		10 (37)	4 (14)	5 (18)	8 (29)	
Positive pulmonary disease history			33 (39)	16 (6)	**< 0.001**	12 (36)	8 (24)	4 (12)	9 (27)	0.46	4 (25)	2 (12)	0	10 (62)	0.13
Married			76 (90)	220 (87)	**0.05**	25 (33)	14 (18)	14 (18)	23 (30)	**0.04**	48 (22)	61 (28)	58 (26)	53 (24)	0.42
Educated^a^			31 (18)	141 (82)	**0.002**	9 (29)	2 (6)	6 (19)	14 (45)	**0.04**	34 (24)	44 (31)	32(23)	31 (22)	0.18
Owning a car			44 (52)	51 (20)	**< 0.001**	10 (22)	6 (14)	9 (20)	19 (43)	**0.02**	11 (21)	9 (18)	6 (12)	25 (49)	**< 0.001**
Owning a home			68 (81)	76 (30)	**< 0.001**	20 (29)	13 (19)	14 (21)	21 (31)	0.7	13 (17)	15 (20)	21 (28)	27 (36)	**0.01**
Employed			18 (21)	29 (11)	**< 0.001**	7 (39)	5 (28)	1 (5)	5 (28)	0.42	6 (20)	10 (34)	7 (24)	6 (21)	**< 0.001**
BMI (kg/m2)			25.6 ± 4.8	25.5 ± 5	0.87	24 ± 4	26 ± 6	26 ± 4	26 ± 6	0.48	25 ± 3	26 ± 4	25 ± 3	26 ± 4	0.22
Physical activity (MET-min/wk)			5284.8 ± 8097	10727.2 ± 6709.9	**< 0.001**	5892 ± 8781	5499 ± 10,480	4899 ± 5979	4736 ± 7165	0.96	11,514 ± 5500	12,896 ± 6090	8948 ± 7264	9516 ± 7105	**0.002**
Smoking Status	Active smoker		48 (57)	104 (41)	**< 0.001**	13 (27)	7 (15)	8 (16)	20 (41)	**0.05**	28 (27)	33 (32)	22 (21)	21 (20)	**< 0.001**
	Ex-smoker		0	51 (20)	**< 0.001**	0	0	0	0		15 (29)	18 (35)	14 (27)	4 (8)	
	Non-smoker		36 (43)	77 (30)	**< 0.001**	14 (39)	9 (25)	8 (22)	5 (14)		12 (16)	17 (22)	14 (18)	34 (44)	
Using waterpipe			4 (5)	10 (4)	**0.024**	1 (25)	1 (25)	1 (25)	1 (25)	0.56	4 (40)	2 (20)	1 (10)	3 (30)	**< 0.001**
Passive smoker			14 (17)	10 (4)	**< 0.001**	5 (36)	3 (21)	3 (21)	3 (21)	0.9	3 (30)	1 (10)	1 (10)	5 (50)	**< 0.001**

Variables: Age, BMI and physical activity presents means ± SD while other variable values are counts. Values between parentheses are percentages

*p* values were calculated from independent Student’s t test, one-way ANOVA or χ2 test

*BMI* body mass index

^a^Educated: having at least finished high school

Table 2 presents the disease severity characteristics of the cases in the study. As expected [48], cases were more likely to have phlegm, cough, while controls were more likely to have higher FEV1% predicted value, FVC and FEV1/FVC ratio. No other significant results were reached in terms of disease severity characteristics across quartiles of HEI-2010 score.

**Table 2:** Characteristics of study participants based on disease severity

Characteristics		Cases (*n* = 84)	Control (*n* = 252)	Quartiles of HEI-2010 score									
				Cases					Control				
				Q1 (*n* = 27)	Q2 (*n* = 16)	Q3 (*n* = 16)	Q4 (*n* = 25)	*P*-value	Q1 (*n* = 57)	Q2 (*n* = 68)	Q3 (*n* = 68)	Q4 (*n* = 59)	*P*-value
Disease severity	Breath shortness	61.6 ± 21.9	4.1 ± 11.4	66 ± 17	61 ± 22	59 ± 24	59 ± 26	0.66	14 ± 19	20 ± 21	15 ± 19	9 ± 12	0.16
	Phlegm	73 (87)	9 (3)	1.2 ± 0.2	1.2 ± 0.4	1.2 ± 0.4	1.1 ± 0.3	0.29	1.8 ± 0.3	1.8 ± 0.3	1.9 ± 0.3	1.9 ± 0.3	0.77
	Cough	64 (76)	3 (1)	1.2 ± 0.4	1.2 ± 0.4	1.2 ± 0.4	1.2 ± 0.5	0.68	1.9 ± 0.3	1.8 ± 0.3	2 ± 0	2 ± 0	0.17
	FEV1% predicted value	55.2 ± 18.6	94.89 ± 12.44	51 ± 18	53 ± 18	63 ± 19	55 ± 18	0.21	91 ± 13	97 ± 13	96 ± 8	95 ± 13	0.49
	FVC	71.2 ± 17.9	92.70 ± 13.21	67 ± 14	71 ± 19	83 ± 16	67 ± 19	0.02	86 ± 15	94 ± 16	92 ± 9	94 ± 13	0.37
	FEV1/FVC ratio	62.7 ± 9.3	82.41 ± 6.07	60 ± 10	62 ± 9	63 ± 9	64 ± 9	0.59	84 ± 8	84 ± 6	83 ± 5	80 ± 5	0.13

Breath shortness, FEV1% predicted value, FVC, FEV1/FVC ratio presents means ± SD while other variable values are counts. Values between parentheses are percentages

*p* values were calculated from independent Student’s t test, one-way ANOVA or χ2 test

*FEV1%* Forced expiratory volume in the first second, *FVC* forced vital capacity, *FEV1/FVC ratio* ratio of the forced expiratory volume in the first one second to the forced vital capacity of the lungs

Dietary intakes of cases and controls across the quartiles of HEI-2010 score are presented in Table 3. Cases had higher intakes of total energy, carbohydrates, and vitamin A compared to controls. On the food groups level of HEI-2010 index, participants with COPD had greater intakes of dairy, empty calories while having less intakes of sea food and plant proteins and whole grains. Table 3 presents cases and controls compared in relation to their dietary intake across quartiles of HEI-2010 score levels. Cases in the top category of HEI-2010 score had higher intake of energy, protein, carbohydrates, fiber, and fat compared with participants in the bottom category of HEI-2010 score. In addition, cases in the top quartile of HEI-2010 had higher intakes of calcium, riboflavin, vitamins A, C, and E, thiamin,, and folate compared to individuals in the bottom category. They had also higher intakes of vegetables, whole fruits, dairy, seafood and plant proteins, ,and whole grain compared to those in the lowest category. As for controls, subjects in the highest quartile of HEI-2010 score had higher intakes of energy, carbohydrates, fiber and fats compared to those in the lowest quartile of HEI-2010 score. In terms of micronutrients, controls in the top category og HEI-2010 score have higher intakkes of calcium, riboflavin, vitamin A, E and C, thiamin, niacin and folate compared to those in the lowest category of HEI-2010 score. In terms of HEI-2010 food groups, controls in the top category of HEI-2010 score have higher intakes of whole and refined grains, total and whole fruits, vegetables, dairy, total protein foods and sea food and plant proteins compared to those in the bottom category of HEI-2010 score.

**Table 3:** Dietary intakes of study participants

	Cases (*n* = 84)	Control (*n* = 252)	*P*-value	HEI-2010 score Quartiles									
				Cases					Control				
				Q1 (*n* = 27)	Q2 (*n* = 16)	Q3 (*n* = 16)	Q4 (*n* = 25)	*P*-value	Q1 (*n* = 57)	Q2 (*n* = 68)	Q3 (*n* = 68)	Q4 (*n* = 59)	*P*-value
Energy (Kcal)	2882 ± 781	2530 ± 829	**0.001**	2594 ± 842	2880 ± 723	2750 ± 639	3277 ± 700	**0.01**	2271 ± 730	2569 ± 471	2544 ± 597	3102 ± 682	**< 0.001**
**Macronutrients**													
Protein (g/day)	95 ± 25	108 ± 158	0.22	82 ± 23	96 ± 22	93 ± 18	109 ± 24	**0.001**	144 ± 331	94 ± 15	96 ± 23	111 ± 23	0.28
Carbohydrate (g/day)	462 ± 137	415 ± 128	**0.005**	421 ± 154	457 ± 131	438 ± 99	524 ± 125	**0.04**	368 ± 165	422 ± 82	380 ± 118	492 ± 104	**< 0.001**
Fiber (g/day)	19 ± 7	20 ± 11	0.38	14 ± 5	17 ± 3	20 ± 4	25 ± 8	**< 0.001**	18 ± 11	21 ± 8	21 ± 10	26 ± 7	**< 0.001**
Fats (g/day)	78 ± 30	70 ± 35	0.07	67 ± 31	78 ± 26	74 ± 30	89 ± 29	**0.06**	61 ± 49	62 ± 21	73 ± 32	84 ± 29	**< 0.001**
**Micronutrients**													
Iron (mg/day)	28 ± 10	40 ± 223	0.40	26 ± 10	28 ± 8	25 ± 6	30 ± 11	0.41	108 ± 470	20 ± 7	17 ± 7	25 ± 9	0.09
Calcium (mg/day)	1246 ± 414	1159 ± 489	0.11	934 ± 269	1220 ± 351	1221 ± 201	1614 ± 398	**< 0.001**	995 ± 623	1075 ± 159	1278 ± 512	1398 ± 313	**< 0.001**
Riboflavin (mg/day)	2 ± 1	2 ± 1	0.71	1.8 ± 0.49	2.±0.6	2 ± 0.4	3 ± 0.8	**< 0.001**	2 ± 0.6	2.4 ± 0.4	2.5 ± 0.6	2.7 ± 0.5	**< 0.001**
Vit A (IU/day)	2255 ± 1706	1701 ± 1454	**0.004**	1237 ± 843	1934 ± 1011	2946 ± 2100	3117 ± 1888	**< 0.001**	1109 ± 598	1532 ± 855	1446 ± 1034	2929 ± 2150	**< 0.001**
Vit E (IU/day)	7 ± 4	7 ± 5	0.43	5 ± 4	7 ± 3	9 ± 3	9 ± 4	**0.005**	5 ± 3	6 ± 3	10 ± 8	8 ± 3	**< 0.001**
Thiamin (mg/day)	2 ± 0.7	2.3 ± 0.8	0.11	2 ± 0.6	2 ± 0.7	2 ± 0.4	3 ± 0.7	**0.01**	2 ± 0.5	2 ± 0.6	2 ± 0.8	3 ± 0.6	**< 0.001**
Niacin (mg/day)	28 ± 9	2 ± 9	0.50	26 ± 9	29 ± 8	26 ± 7	31 ± 9	0.22	25 ± 7	29 ± 6	25 ± 9	32 ± 8	**< 0.001**
Folate (mcg/day)	384 ± 139	351 ± 133	0.06	319 ± 140	366 ± 129	395 ± 102	458 ± 133	**0.003**	300 ± 89	361 ± 94	378 ± 110	433 ± 107	**< 0.001**
Vitamin C (mg/day)	144 ± 70	138 ± 66	0.45	85 ± 34	129 ± 38	158 ± 32	208 ± 76	**< 0.001**	97 ± 29	127 ± 30	151 ± 63	197 ± 63	**< 0.001**
**HEI-2010 food groups**													
Whole grain (g/day)	5 ± 14	19 ± 20	**≤ 0.001**	18 ± 31	49 ± 101	44 ± 66	88 ± 126	**0.04**	71 ± 72	128 ± 103	135 ± 95	153 ± 140	**< 0.001**
Refined grains (g/day)	586 ± 192	576 ± 194	0.69	562 ± 174	581 ± 224	542 ± 127	552 ± 202	0.94	537 ± 170	659 ± 246	409 ± 181	606 ± 195	**< 0.001**
Total fruit (g/day)	363 ± 208	354 ± 181	0.67	187 ± 103	322 ± 116	401 ± 147	557 ± 203	**< 0.001**	242 ± 89	308 ± 91	351 ± 156	517 ± 233	**< 0.001**
Whole Fruits (g/day)	369 ± 214	352 ± 182	0.47	168 ± 103	278 ± 95	370 ± 131	505 ± 203	**< 0.001**	224 ± 123	298 ± 112	279 ± 127	514 ± 266	**< 0.001**
Vegetables (g/day)	364 ± 153	338 ± 176	0.53	230 ± 78	315 ± 70	415 ± 114	468 ± 160	**< 0.001**	225 ± 59	290 ± 89	340 ± 84	508 ± 223	**< 0.001**
Dairy (g/day)	501 ± 237	424 ± 224	**0.01**	246 ± 149	340 ± 170	379 ± 196	606 ± 229	**< 0.001**	219 ± 98	323 ± 113	384 ± 91	482 ± 202	**< 0.001**
Total protein foods (g/day)	98 ± 39	101 ± 44	0.52	68 ± 34	67 ± 36	65 ± 41	65 ± 41	0.65	49 ± 57	42 ± 22	56 ± 25	68 ± 40	**0.02**
Sea food and plant protein (g/day)	70 ± 33	89 ± 45	**< 0.001**	54 ± 21	59 ± 16	74 ± 25	91 ± 45	**< 0.001**	65 ± 20	79 ± 19	93 ± 54	100 ± 48	**0.05**
Empty calories (g/day)	141 ± 112	100 ± 78	**≤ 0.001**	138 ± 116	115 ± 63	148 ± 158	147 ± 84	0.79	166 ± 135	137 ± 100	112 ± 114	99 ± 81	0.18

Values presented as mean ± SD, obtained by independent sample t test and ANOVA

Empty calories: Calories that come from solid fats, alcohol, and added sugars are counted. If alcohol consumption exceeds 13 g per 1000 calories, it is included in the count

Whole fruits: This component measures all forms of fruit except juice

Total fruit: This component measures all forms of fruit, including 100% fruit juice

HEI-2010 score and dietary food groups intake of COPD cases according to disease severity are presented in Table 4. Cases in the higher COPD severity level had significantly higher HEI-2010 score and whole grains consumption compared to the lowest COPD severity level. No other significant results were reached in terms of other dietary food groups and COPD severity levels.

**Table 4:** Healthy eating index (HEI-2010) score and dietary food groups intake of the participants according to disease severity of chronic obstructive pulmonary disease

COPD severity levels	Mild (*n *= 7)	Moderate (*n* = 45)	Severe (*n* = 22)	Very severe (*n* = 10)	*P* value
**HEI-score**	1233 ± 253	1359 ± 495	1052 ± 406	1428 ± 472	**0.055**
**Whole grains**	8.2 ± 19.8	60.3 ± 96.7	15.6 ± 27.2	107.8 ± 145.4	**0.02**
**Refined grains (g/d)**	503 ± 212	593 ± 188	557 ± 181	447 ± 83	0.11
**Total fruits (g/d)**	289.7 ± 167.5	411.2 ± 237.3	300 ± 166.7	335.2 ± 179.9	0.15
**Whole fruits (g/d)**	267 ± 161	370 ± 218	275 ± 156	447 ± 83	0.21
**Vegetables (g/d)**	330.7 ± 156.7	369.3 ± 145.6	305.5 ± 148.3	397.6 ± 164.7	0.29
**Dairy (g/d)**	485.9 ± 208.6	393.4 ± 236.2	329.2 ± 197.1	501.5 ± 304.2	0.19
**Total protein foods (g/d)**	69.6 ± 57.6	77.4 ± 40.8	61.2 ± 25.1	53.8 ± 22.6	0.19
**Sea foods and plant proteins (g/d)**	54 ± 36	77 ± 38	60 ± 20	67 ± 25	0.14
**Empty calories (g/d)**	128 ± 81	138 ± 99	150 ± 146	123 ± 71	0.92

Data are presented as mean ± SD

Obtained by ANOVA test

Findings from logistic regression about the association between HEI-2010 and COPD are presented in Table 5. Individuals with more compliance to HEI-2010 recommendations (higher HEI score) had significantly lower odds of COPD (OR: 0.34; 95% CI: 0.16–0.72) in comparison to participants having lower HEI-2010 score. After controlling for possible confounders, individuals with the highest HEI score (having more healthy diet) were 82% less prone to have COPD in relation with participants with lowest HEI score (having less healthy diet) (OR: 0.18; 95% CI: 0.03–0.96). This significant association continued after adjusting for smoking and physical activity (OR: 0.08; 95% CI: 0.01–0.93) and with additional adjustment for BMI (OR: 0.08; 95% CI: 0.01–0.92).

**Table 5:** Association Between Healthy Eating Index (HEI-2010) Score and Risk of chronic obstructive pulmonary disease (COPD)

Healthy eating index score range	1 (***n***= 84)	2 (***n***= 84)		3 (***n***= 87)		4 (***n***= 81)		P trend
	OR	OR	95% CI	OR	95% CI	OR	95% CI	
**Crude**	1.00	0.72	0.38-1.38	0.44	0.22-0.87	0.34	0.16-0.72	**0.002**
**Model I**	1.00	0.66	0.21-2.1	0.34	0.1-1.5	0.18	0.03-0.96	**0.04**
**Model II**	1.00	0.51	0.11-2.24	0.29	0.04-1.98	0.08	0.01-0.93	**0.05**
**Model III**	1.00	0.5	0.11-2.23	0.29	0.04-1.95	0.08	0.01-0.92	**0.046**

Model I: adjusted for energy, age, gender, residence, marital status, employment, owning a car/home, education, family history of COPD

Model II: Further adjusted for smoking and physical activity

Model III: Further adjusted for BMI

## Discussion

In this case-control study, we found a significant inverse association between adherence to HEI-2010 recommendations and odds of COPD. This association remained significant after adjusting for several confounding variables. As far as we know, this study is the earliest to examine the association between HEI-2010 and the odds of COPD in a Middle Eastern region.

Chronic Obstructive Pulmonary Disease (COPD) is a significant contributor to morbidity and mortality on a global scale. Our study found that adherence to the HEI-2010 guidelines was linked to a decreased likelihood of COPD. These findings corroborate previous studies that have reported an inverse relationship between healthy dietary patterns and the incidence of COPD [19–21, 24, 26, 49–53]. Several studies have investigated the relationship between dietary patterns and the risk of COPD. For instance, a cross-sectional study of American adults found that a higher Dietary Approaches to Stop Hypertension (DASH) diet score was associated with a lower risk of COPD [24] Similarly, a case-control study in Iranian adults reported an inverse association between adherence to a low-carbohydrate diet (LCD) and the odds of COPD [26]. In an Asian population cohort, adherence to multiple recommended dietary patterns was associated with a 14–28% lower risk of death from respiratory diseases [50]. Similarly, the Swiss Cohort Study on Air Pollution and Lung and Heart Diseases in Adults (SAPALDIA) found a protective association between consumption of a diet rich in fruits, vegetables, fish, and nuts and age-related chronic respiratory disease [49]. In contrast, a Chinese population cohort reported that a Western-like dietary pattern was associated with an increased incidence of cough with phlegm [51]. In addition, a study done in Iran found an association between decreased scores of HEI and Mediterranean diet indices and severity of COPD symptoms, but results were not statistically significant [54]. In the mentioned study, the author investigated the association between adherence to healthy dietary patterns and severity of COPD symptoms. In contrast, our current study assessed the association between adhering to HEI-2010 dietary guidelines and the odds of COPD. An important factor considered in this study is the use of HEI-2010 index as a healthy reference for dietary pattern, whereas previous studies used different dietary patterns. Another difference is the low prevalence of smoking among Iranian women compared to other countries [55]. Overall, these findings suggest that healthy dietary intakes may be protectively associated with COPD and its symptoms. However, further studies in developing countries are required to reach firm conclusions.

After prolonged exposure to risk factors like tobacco smoke and air pollution, the primary mechanisms that cause COPD are oxidative stress and inflammation in the lungs and bloodstream [56]. Dietary factors play an important role in improving inflammation, oxidative stress and the regulation of immunity and metabolism [57]. As a result, we hypothesize that the nutritious ingredients in the HEI-2010 diet could potentially hinder or postpone the development of COPD. Additionally, dietary factors may be involved in the modification of the gut microbiota, which can enhance the immunity, metabolism and inflammation by producing short chain fatty acids (SCFAs), considered important active metabolites [57]. Other studies have concluded that diets rich in high-fiber plants, influenced the gut microbiota which enhance the circulation levels of SCFA which in turn have an anti-inflammatory effect. In contrast, diets rich in animal products, which are high in choline and carnitine, have been linked to increased levels of Trimethylamine N-oxide (TMAO), associated with the risk of cardiovascular disorders, atherosclerosis, and death [58]. High dietary fiber intake has been linked to increased levels of SCFAs, which have a protective effect against inflammatory airway disease due to their anti-inflammatory properties [59]. while TMAO levels have been associated with mortality in COPD patients [60]. An evolving concept is the gut-lung axis, where modulation of intestinal microbiota can aid in the management of respiratory diseases [61]. SCFAs have been found in sputum, indicating a link between the lungs and the intestine [62]. They help maintain lung immune-metabolic tone and can act on various parts of the innate immune defenses of the lungs [63]. Butyrate and propionate may help restore and maintain the barrier function of damaged airway epithelium by increasing the expression of ZO-1 tight junction proteins [64]. This is important because airway epithelial barrier dysfunction and impaired tight cell contacts can develop in smoking and COPD [65]. Therefore, SCFAs may have clinical significance in regulating barrier function [64].

Some strengths of the current study include the use of a healthy dietary index rather than individual components of a healthy diet, the use of a validated food frequency questionnaire (FFQ), adjustment for several confounders, and being the first study in a Middle Eastern population to assess the relationship between the HEI-2010 and the risk of COPD. Our study has some limitations that should be considered when interpreting the results. One limitation is the significant differences between the case and control groups in terms of age, marital status, education level, social and economic status, physical activity level, and smoking status. While we adjusted for these variables in our analysis using logistic regression and matched the case and control groups based on age and sex, these differences could still potentially impact our results. Another limitation of our study is the potential residual confounding due to smoking. Despite our efforts to control its effect by adjusting for its confounding effect in logistic regression analysis, it is possible that some confounding remains. Future studies may consider using matching based on smoking status or other methods to better control the confounding effect of smoking. This can help to improve the validity and reliability of the results. Another limitation of our study is the substantial differences in energy consumption and other micronutrient intakes between our case and control groups. While we attempted to account for these differences through adjustment, this method may not completely eliminate their effects. Additionally, our sample size was not large enough to support a stratified analysis based on energy intake levels. As such, the observed associations between dietary intakes and the outcome of interest may be influenced by these differences. Also, The HEI-2010 is an index based on dietary patterns in the USA and may not be applicable in other countries with different dietary habits. Nevertheless, the validity of the HEI-2010 index has been demonstrated in a previous study conducted in an Iranian population where it detected an association with the risk of breast cancer [66]. The case-control design of the study prevents us from drawing conclusions about causality due to selection and recall bias. Measurement errors may result in misclassification of study participants. Given the sample size of the current study, our findings should be generalized with caution.

## Conclusion

In our case-control study, we found a significant inverse association between adherence to the HEI-2010 recommendations and the odds of developing COPD in a Middle Eastern population. This association remained significant even after adjusting for several confounding variables. To our knowledge, this is the first study to examine this association in a Middle Eastern region. While previous research has shown that healthy dietary habits can reduce the risk of developing COPD, our study supports these findings by demonstrating that adherence to the HEI-2010 guidelines is associated with a decreased likelihood of COPD. These results are consistent with other studies that have reported an inverse relationship between healthy dietary patterns and the incidence of COPD. Overall, our findings suggest that following a healthy diet, as recommended by the HEI-2010 guidelines, may play an important role in reducing the risk of COPD and improving lung function. Further research is needed to confirm these findings and to better understand the underlying mechanisms of this association.

### Supplementary Information

**Additional file 1.**

## Data Availability

The datasets generated during and analyzed during the current study are not publicly available due to ethical concerns but are available from the corresponding author on reasonable request.

## Abbreviations

COPD: Chronic obstructive pulmonary disease
HEI-2010: Healthy Eating index 2010
SES: Social economic status
FFQ: Food frequency questionnaire
BMI: Body mass index
SCFA: Short chain fatty acids
TMAO: Trimethylamine N-oxide
PUFAs: Polyunsaturated fatty acids
MUFAs: Monounsaturated fatty acids
CI: Confidence Interval
OR: Odd Ratio
SD: Standard Deviation
ANOVA: One-way analysis of variance
