# Supplementary material for: The association between diet quality and chronic obstructive pulmonary disease: a case-control study
Source: BMC Public Health. 2023 Oct 23;23:2071. doi: 10.1186/s12889-023-16586-8 (PMC10591368; doi:10.1186/s12889-023-16586-8)
Supplement: Supplementary file 1 — Additional file 1. [file 12889_2023_16586_MOESM1_ESM.docx]

**Target population**: COPD patients.

**Sampling frame**: Patients from Al-Zahra referral hospital- Isfahan

**Cases**: Patients aged >30 years diagnosed with **Controls**: Patients visiting the outpatient

COPD by a pulmonologist (FEV1/FVC < 70% or clinics in the same hospital as cases.

FEV1 < 80%) through spirometry test.

**Matching**: Cases and controls matched by age (range 5 years) and sex, on individual-by-individual basis.

**Inclusion criteria**: Diagnosed with COPD based on physician diagnosis and spirometry test.

**Non-inclusion criteria**: History of stroke, dementia, or any health condition preventing interview; renal failure, inflammatory bowel disease, long-term steroid treatment, severe heart failure, chronic infections (tuberculosis, HIV etc.), chronic liver cirrhosis, cachexia, uncontrolled thyroid disease, rheumatoid arthritis, cancer in the past 3 years and other pulmonary problems.

**Sampling method**: Simple random sampling

**Final sample**: 84 cases and 252 controls

**Supplementary figure: Sampling diagram for the study population.**
